# Supplementary material for: Chromothripsis during telomere crisis is independent of NHEJ, and consistent with a replicative origin
Source: Genome Res. 2019 May;29(5):737–49. doi: 10.1101/gr.240705.118 (PMC6499312; doi:10.1101/gr.240705.118)
Supplement: Supplemental Material [file supp_gr.240705.118_Supplemental_file_1.zip › contigs/annotated_contigs/DB41/contig.2.DB41_length_754_mean_cov_8.32360742706.docx]

**DB41_length_754_mean_cov_8.32360742706**

```
TGAGGATGATTTGCATAATTCATTTTTAGAACATTAATTAGCCGGGATGTGTAATAGTTCTTGTGCCAATTTGTGTTTCAATTTCAAAT
 >chr2:16255564-16255919 + E=9e-199
GCAAATACAAATTAGCAAATGTTATTCCTGTGGCACCTTAAGGATGAAACATTTGCTGCTCGTCGTATTAATCTTTACATTTCTGAAAC

TATCACTGATAGAAGCAATAATATCCTCTAATTTGCAGGATTGAGAAAAAAATGTAGTCTGATGGGGGCTGGGGTGGGTGAGAGGTTAA

TTCTCTTTCAGCAAAAGAAAACAGCATCCATCCATCGATTAAATAGTGCTGAGTACCTACTAAATGTCAGACGTTGTGGTAGCAG|GCA
                                                                                      >c
|TACTGGCTCTGCTCCTGCAACTGAAGCAGACATCAGGACCCGGTCTGTTTGAGCCAGAAACAAAGGGCACCAAGCAATAAAAATGAGC
hr17:75869818-75870220 + E=5e-230
CCATCTTCTCGTCCCACACCTCCCACGGCCATGGCCTCACAGGAGCCCCGTCTCCTCGCTGAATACGTCCAAGGATGTGTCCATTTCAT

CCTGGCTGTTTTAAAAAGCCATCCTGGAAAAGATGCTCACGTGGGTAGCCCTTTGCCTCAGCGTCAGCTGGTTGACTCCACTCGAAGCA

GCCCCTTCCTGCACCAGTAGCCCCTCCACCCTGCTCAAACCCGAAACTGGGGTCCCCTTCAAGGTCTCTGTGCCCCTTGCCCCAGGCCA

AGCCTGCAGAGTCCAGCCTTGCAGACCAAGCCTGCAAAGCCCAT
```
